# Predictive factors for engraftment kinetics of autologous hematopoietic stem cells in children

**DOI:** 10.1016/j.htct.2024.09.2481

**Published:** 2024-11-07

**Authors:** Biljana Andrić, Dragana Vujić, Olivera Šerbić, Zorica Radonjić, Marija Simić, Miloš Kuzmanović

**Affiliations:** a Mother and Child Health Care Institute of Serbia “Dr. Vukan Čupić”, Belgrade, Serbia; b Faculty of Medicine, University of Belgrade, Belgrade, Serbia

**Keywords:** Hematopoietic stem cell transplantation, Engraftment, Predictive factors, Children

## Abstract

**Background:**

Engraftment after hematopoietic stem cell transplantation is the recovery rate of neutrophils and platelets. This study aimed to test the impact of the patient's general characteristics, pre-transplantation factors, and quality parameters of hematopietic stem cell products on hematopietic recovery and to define predictive factors for engraftment in children.

**Methods:**

This retrospective study included 52 patients aged from 1 to 18 years old treated with autologous transplantation at the Mother and Child Health Care Institute of Serbia “Dr. Vukan Čupić” in Belgrade, from January 2013 until December 2018. Data were collected from medical records and apheresis procedure protocols. SPSS 20.0 software package was used for statistical data processing.

**Results:**

The median neutrophil engraftment was 18.0 (16.0–22.5) days, while the median platelet engraftment was 11.0 (10.0–18.0) days. Statistically significant correlations were found between neutrophil engraftment and patient's age (*p*-value = 0.050), body weight (*p*-value = 0.021), diagnosis (*p*-value = 0.023), source of stem cells (p-value = 0.001), and the number of CFU-GM/kg (*p*-value = 0.018). A statistically significant correlation was found between platelet engraftment and the time from diagnosis to the transplantation (*p*-value = 0.043), source of stem cells (p-value = 0.009), and the number of CD34^+^ cells/kg (*p*-value = 0.014).

**Conclusions:**

Predictive factors for hematopoietic recovery in this study were the patient's age, body weight, diagnosis, time from diagnosis to hematopoietic stem cell transplantation, source of hematopietic stem cells, the number of CD34^+^ cells/kg, and the number of CFU-GM/kg.

## Introduction

According to the recommendations of the European Society for Blood and Marrow Transplantation (EBMT) and according to protocols for the treatment of childhood cancer, high-dose chemotherapy followed by autologous hematopoietic stem cell transplantation (HSCT) is used as a therapy in high-risk forms of some solid tumors and refractory/relapsing lymphomas. Indications for autologous HSCT in children and adolescents include neuroblastomas (high-risk or first complete remission), Ewing sarcoma (localized disease at the time of diagnosis, tumor volume less than 200 mL), retinoblastoma (high-risk or relapse), brain tumors (high-risk, children under three years of age or in all cases of relapse), Wilms’ tumor in relapse, yolk Sac tumor in relapse, Hodgkin's lymphoma (poor response to first-line therapy or relapse), and non-Hodgkin B lymphoma (initially poor response or relapse). Treatment of other solid tumors and autoimmune diseases with autologous HSCT is mainly done through clinical studies.^1^, ^2^, ^3^, ^4^, ^5^

A high dose of chemotherapy - in cases where it had not been previously used - enables consolidation and remission, thus overcoming therapeutic resistance. The consequent infusion of autologous hematopoietic stem cells (HSCs) enables hematopoietic system recovery.

Two of the most commonly used indicators of hematopoietic reconstitution are rates of neutrophil and platelet cell line recoveries. Neutrophil engraftment is most widely defined as the first of three consecutive days with a sustained absolute neutrophil count (ANC) above 0.5 × 10^9^/L. Platelet engraftment is independent of platelet transfusion for at least seven days with a platelet count exceeding 20 × 10^9^/L.^6^^,^^7^ When engraftment is achieved, autonomous hematopoiesis has recommenced.

This study aimed to quantify the impact of general patient characteristics, pre-transplantation factors, and quality parameters of HSC products on hematopoietic recovery, considering overall recovery kinetics and establishing predictive factors for engraftment of autologous HSCs in children and, with that, possible prediction of therapeutic outcome.

## Material and methods

This retrospective study included 52 patients aged 1 to 18 years treated with autologous HSCT at the Mother and Child Health Care Institute of Serbia “Dr. Vukan Čupić” from January 2013 until December 2018.

Peripheral HSCs (CD34^+^) were collected using continuous flow cell separators: Cobe Spectra, version 6.1; (Gambro BCT, Lakewood, CO, United States) and Spectra Optia version 11 (Terumo BCT, United States).

In patients weighing less than 25 kg, the HSC collection system was filled with irradiated allogenic red blood cells (RBCs). Before the procedure started, the hemoglobin value had to be >8.0 g/dL, while the platelet count could not be below 30 × 10^9^/L. All patients had at least two and, optimally, three vascular accesses. Acid citrate dextrose-A (ACD-A) solution was used as an anticoagulant during the apheresis procedure. The patients were monitored for ionized calcium before and during HSC apheresis with the ionized calcium values being maintained above 1 mmol/L using dissolved effervescent calcium tablets *per os* at a dose of 0.5 g/10 kg.

In patients who were poor mobilizers for peripheral blood stem cells, the source of HSCs was bone marrow (BM) as plerixafor was not approved for use in pediatric patients in the Republic of Serbia. Bone marrow was collected from the superior and posterior crest of the iliac bone using a special needle for BM harvesting (Biomedical, Florence, Italy). A specialized closed-circuit collection system containing multiple filters was used to collect BM (BioAccess, Contract Medical International GmbH, Dresden, Germany). Heparin was used as an anticoagulant. To reduce the number of RBCs in the product before cryopreservation, the BM product was processed using continuous flow cell separators: Cobe Spectra and Spectra Optia after harvesting. FACSCalibur flow cytometer and FACSCanto II (Beckton Dickinson, USA) were used for CD34^+^ cell counting. Paint-a-gate and Diva software were used for multiparameter analysis. The HSC product was cryopreserved in a controlled rate freezer (IceCube 14S; Sy-Lab, Neupurkersdorf, Austria).

The concentration of hemoglobin and hematocrit in peripheral blood and HSC products was determined by automatic hematology counters: ADVIA 2010 (Siemens, Germany) and Sysmex XN-1000 (Sysmex Europe GmbH, Germany). Patients' ionized calcium was determined using a RAPIDPoint 500 gas analyzer (Siemens, Germany).

Descriptive and analytical statistical methods were used for statistical data processing. The descriptive statistical methods used are absolute (n) and relative numbers (%), measures of central tendency (median), and measures of dispersion (range, percentiles). Nonparametric difference tests, such as the Mann-Whitney U and Kruskal-Wallis tests, were used for statistical analysis. Spearman's correlation analysis was used to analyze the correlation. The Statistical Package for Social Sciences (SPSS) version 20.0 (IBM Corp., Armonk, NY) software package and R version 3.4.2 (R Core Team, Vienna, Austria.) were used for statistical data analysis. A *p*-value ≤0.05 was considered statistically significant.

## Results

Table 1 shows the general characteristics of the 52 patients in the entire cohort (age, body height, body weight, gender, diagnosis).

**Table 1:** Patients’ general characteristics and pre-transplantation factors.

Variable - (*n* = 52)	
Age (years) - Median (range)	7.29 (1.1–18.7)
≥10 - n (%)	24 (46.2)
<10 - n (%)	28 (53.8)
Body height (cm) - Median (range)	121.0 (81.0–191.0)
Body weight (kg) - Median (range)	23.7 (7.3–101.8)
≥20 - n (%)	28 (53.8)
<20 - n (%)	24 (46.2)
Gender - n (%)	
Male	27 (51.9)
Female	25 (48.1)
Diagnosis - n (%)	
Neuroblastoma	24 (46.2)
Ewing sarcoma	18 (34.6)
Hodgkin lymphoma	7 (13.5)
Non-Hodgkin lymphoma	2 (3.8)
Medulloblastoma	1 (1.9)
Time from diagnosis to HSCT (months) - Median (range)	8 (4.0–32.0)
≥10 - n (%)	21 (40.4)
<10 - n (%)	31 (59.6)

HSCT – hematopoietic stem cell transplantation.

The patients were divided into two subgroups: under 10-year-old and over 10-year-old. According to body weight, the patients were divided into those with less than 20 kg of body weight and children with ≥20 kg. Pre-transplantation factors, diagnosis, and the time from diagnosis to HSCT (in months) are also included in Table 1.

Table 2 shows the source of HSCs and the quality parameters of HSC products (the number of CD34^+^ cells/kg, mononuclear cells [MNC]/kg, and colony forming unit-granulocytes macrophage [CFU-GM]/kg).

**Table 2:** Source of HSCs and quality parameters of HSC product.

Parameter (n = 52)	
The source of HSCs - n (%)	
Peripheral blood	44 (84.6)
Bone marrow	5 (9.6)
Both	3 (5.8)
CD34^+^ cells/kg (x 10^6^) - Median (range)	6.1 (2.01–15.3)
≥6 - n (%)	29 (55.8)
<6 - n (%)	23 (44.2)
MNC/kg (x10^8^) - Median (range)	4.5 (0.26–41.0)
CFU-GM/kg (x10^4^) - Median (range)	43.3 (0.7–398.0)
≥50 - n (%)	22 (42.3)
<50 - n (%)	30 (57.7)

HSCs: hematopoietic stem cells; MNC: mononuclear cells; CFU-GM: colony forming unit- granulocyte macrophages.

The group of patients was divided into two subgroups according to the median number of infused CD34^+^ cells/kg (<6 and ≥6 × 10^6^ CD34^+^ cells/kg). According to the number of CFU-GM/kg, the patient group was divided into subgroups with less than 50 and ≥50 × 10^4^ CFU-GM/kg (Table 2).

**Figure 1:**
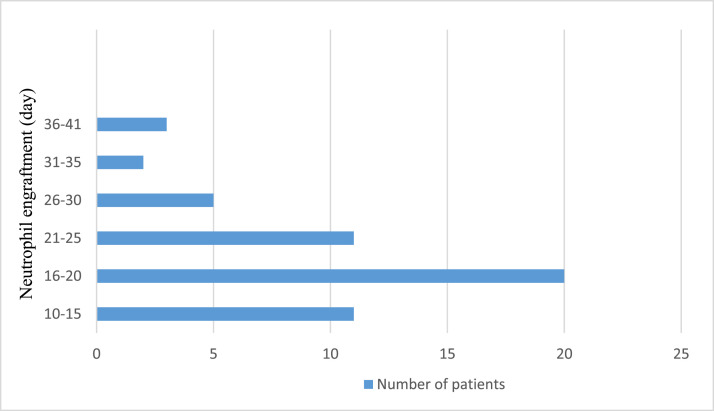
Neutrophil engraftment. The horizontal line shows the number of patients, and the vertical line shows the consecutive number of days when neutrophil engraftment was achieved.

**Figure 2:**
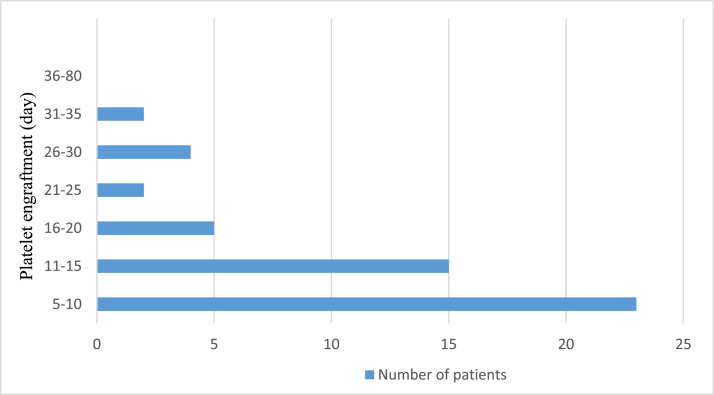
Platelet engraftment. The horizontal line shows the number of patients, and the vertical line shows the consecutive days when platelet engraftment was achieved.

Neutrophil engraftment was achieved at a median of 18 days (range: 16.0–22.5 days). Figure 1 shows the neutrophil engraftment of the entire patient cohort. On the other hand, platelet engraftment was reached at a median of 11 days (range: 10.0–18.0 days). Figure 2 shows the platelet engraftment of the entire patient cohort.

A comparison of general patients’ characteristics, pre-transplantation factors, and quality parameters of the HSC product as predictive factors for neutrophil and platelet engraftment (in days) is presented in Table 3.

**Table 3:** Comparison of neutrophil and platelet engraftment with patients’ general characteristics, pre-transplantation factors, and quality parameters of HSC product.

Characteristic	Neutrophil engraftment (day)	*p*-value	Platelet engraftment (day)	*p*-value
Age (year)				
≥10	17.5 (15.8–19.5)		10.5 (9.0–13.3)	
<10	21.0 (16.8–25.3)	**0.050**	11.0 (10.0–21.3)	0.112
Body weight (kg)				
≥20	17.5 (15.8–21.0)		11.0 (9.0 −13.3)	
<20	21.0 (18.8–25.3)	**0.050**	11.5 (10.0–21.3)	0.122
Diagnosis				
NB	20.5 (16.5–26.5)		12.5 (10.0–26.5)	
ES	18.0 (17.0–21.0)	**0.023**	11.5 (9.0–13.0)	0.053
HL	12.0 (11.0–14.0)		9.0 (9.0–11.0)	
Other	19.0 (18.0–21.0)		11.0 (9.0–17.0)	
The time from diagnosis to HSCT (months)				
≥10	18.0 (14.0–21.0)		10.0 (9.0–13.0)	
<10	20.0 (17.0–24.0)	0.339	12.0 (10.0–22.5)	**0.012**
The source of HSCs				
PB	18.0 (15.5–21.0)		10.5 (9.5–13.0)	
BM	33.0 (28.0–36.0)		32.0 (27.0–32.0)	
PB+BM	29.0 (16.0–32.0)	**0.001**	13.0 (10.0–20.0)	**0.009**
CD34+ cells/kg				
(x10^6^)				
≥6	18.0 (15.0–21.0)		10.0 (9.0–12.0)	
<6	21.0 (17.5–25.5)	0.134	13.0 (10.0–25.0)	**0.016**
CFU-GM/kg (x10^4^)				
≥50	17.5 (15.3–18.8)		10.5 (10.0–22.8)	
<50	21.0 (20.0–27.8)	**0.003**	12.0 (10.0–16.3)	0.978

Results are presented as median (25–75th percentile), and *p*-value: bolded numbers are statistically significant. HL: Hodgkin's lymphoma; NHL: non-Hodgkin lymphoma; NB: neuroblastoma; ES: Ewing sarcoma; HSCT: hematopoietic stem cell transplantation; HSC: hematopoietic stem cell; PB: peripheral blood; BM: bone marrow; CFU-GM: colony forming unit-granulocytes macrophage.

The Kruskal-Wallis test showed that diagnosis was a statistically significant predictor for neutrophil engraftment. Bonferroni correction for multiple comparisons showed a statistically significant difference in neutrophil engraftment between patients with Hodgkin's lymphoma and Ewing sarcoma (*p*-value = 0.017) and between patients with Hodgkin's lymphoma and neuroblastoma (*p*-value = 0.002). For platelet engraftment, there was a statistically significant difference between patients with Hodgkin's lymphoma and neuroblastoma (*p*-value = 0.012).

The statistical significance for neutrophil engraftment was not found for body height (*p*-value = 0.081), gender (*p*-value = 0.949), and MNC/kg (*p*-value = 0.110) as predictive factors. Also, statistical significance was not found for body height (*p*-value = 0.109), gender (*p*-value = 0.839), and MNC/kg (*p*-value = 0.862) for platelet engraftment.

Using correlation analysis (Spearmen's rank correlation coefficient), a statistically significant, medium-strong, and negative correlation was found only between body weight and neutrophil engraftment (*r* = −0.319; *p*-value = 0.021). In addition, a weak and negative statistically significant correlation was proven between the time from diagnosis to HSCT and platelet engraftment (*r* = −0.282; *p*-value = 0.043).

There was a medium-strong and negative statistically significant correlation between the number of CD34^+^ cells/kg and platelet engraftment (*r* = −0.337; *p*-value = 0.014) and between the number of CFU-GM/kg and neutrophil engraftment (*r* = −0.327; *p*-value = 0.018). This means engraftment is faster when the CD34^+^ cells/kg and CFU-GM/kg counts are higher.

## Discussion

Transplanted HSCs migrate and settle in bone marrow niches – a process called the ‘homing’ of HSCs. This process is followed by self-renewal and differentiation of HSCs to all blood cell lines, representing the beginning of hematopoietic recovery.^8^ Many factors, including the pre- and post-transplant period, affect the rate of hematopoietic recovery after HSCT.

The current study evaluated the influence of the patient's general characteristics, pre-transplantation factors and the quality of the HSC product on the rate of hematopoietic recovery to define predictive factors for the engraftment of autologous HSCs in children.

In this study the median time to neutrophil engraftment was 18.0 days (range: 16.0–22.5 days), while the median time to platelet engraftment was 11.0 days (10.0–18.0 days). These results are similar to those of two groups of researchers, in which the time of engraftment of platelets was shorter than the engraftment of neutrophils.^9^^,^^10^ In other reports, the median time to neutrophil engraftment was shorter than that of platelet engraftment.^11^, ^12^, ^13^, ^14^, ^15^, ^16^, ^17^, ^18^, ^19^

## Association of patients’ general characteristics with engraftment kinetics

The variables significantly associated with hematopoietic recovery in this study were patient age, body weight, and diagnosis. Patients older than ten years had faster neutrophil recovery than those younger than ten; most of the under 10-year-old were neuroblastoma patients. The current standard therapeutic approach for high-risk neuroblastoma patients consists of intense, aggressive chemotherapy protocols, which lead to BM depletion and, thus, slower hematopoietic recovery after HSCT.

The scarce literature data refer to factors influencing hematopoietic recovery after autologous HSCT in children. Díaz et al. enrolled 46 pediatric patients with hematologic malignancies or solid tumors in their study and the influence of different variables on engraftment kinetics was examined. Variables included patient age, sex, body weight, diagnosis, remission or relapse status at mobilization, transplant conditioning regimens, CFU-GM and CD34^+^ cells/kg infused, duration of previous chemotherapy, and irradiation. They found a significant association between age and neutrophil and platelet engraftment.^11^ Most other studies examining the influence of patients’ general characteristics on engraftment kinetics refer to adult patients. In a study of 70 patients with multiple myeloma, Hodgkin's lymphoma or non-Hodgkin lymphoma, Hassan et al. showed that platelet recovery was faster in patients younger than 50.^9^ The study by Grubović et al. included 90 patients with acute myeloid leukemia, lymphoma, and multiple myeloma; age was shown to have a statistically significant association with neutrophil and platelet recovery in patients with lymphoma treated with autologous HSCT ^6^. A study by Gonçalves et al. testing the influence of gender, age, diagnosis, source of HSCs, and conditioning regimen showed that patients aged 50–59 years had faster neutrophil and platelet recovery than younger patients.^12^ Yamaguchi et al. enrolled 144 patients with non-Hodgkin lymphoma treated with autologous HSCT and tested factors influencing delayed engraftment. This study showed that the patient's age was significantly correlated, albeit borderline, with platelet engraftment.^13^

The current study demonstrated a statistically significant correlation between body weight and neutrophil engraftment. Children weighing ≥20 kg had faster neutrophil engraftment than those weighing <20 kgs. A possible reason may be that the children weighing <20 kg were mainly neuroblastoma patients. Díaz et al. also demonstrated a statistically significant correlation between patient body weight and neutrophil and platelet engraftment*.*^11^ In their study, Hassan et al. concluded that patients weighing ≥60 kg had faster recovery of neutrophils and platelets than patients weighing <60 kg.^9^ Lutfi et al. tested the correlation between patient body weight and delayed engraftment and proved that patients with higher body weight were less likely to have delayed engraftment.^20^

On testing the correlation between gender and hematopoietic recovery in this study, no statistically significant correlation was demonstrated between gender and platelet and neutrophil engraftment. These data correspond to most other authors’ results.^11^^,^^12^^,^^18^, ^19^, ^20^, ^21^, ^22^ On the other hand, a retrospective study by Hillier et al., which included 54 high-risk neuroblastoma patients treated with autologous HSCT, proved an influence of gender on hematopoietic recovery; females had a longer hematopoietic recovery compared to males.^23^

In the present study, we also observed a statistically significant correlation between the diagnosis and neutrophil engraftment, while no statistically significant correlation was found between the diagnosis and platelet engraftment. Significantly faster neutrophil recovery was seen in patients with Hodgkin's lymphoma compared to patients with neuroblastoma and Ewing sarcoma.

Literature data referring to the association of diagnosis with hematopoietic recovery after autologous HSCT are, in most cases, related to adult patients. The only pediatric study was by Díaz et al.; it examined the influence of diagnosis on hematopoietic recovery and demonstrated no significant association between diagnosis and engraftment kinetics.^11^ Hassan et al. proved a statistically significant correlation between diagnosis and neutrophil engraftment. Patients with multiple myeloma had a faster neutrophil recovery than patients with lymphoma.^9^ In a study by Gonçalves et al. that included 65 patients, 25 were treated with autologous HSCT and 40 were treated with allogeneic HSCT. This study showed that patients with multiple myeloma and lymphoma had faster neutrophil recovery than patients with leukemia, myelodysplastic syndrome, and aplastic anemia.^12^

## Association between pre-transplant factors and engraftment kinetics

One possible predictive factor for engraftment kinetics is the time from diagnosis to HSCT. In the study of Ergene et al., a statistically significant correlation was proven between the time from diagnosis to HSCT and the rate of neutrophil recovery; this was confirmed in the study of Grubović et al.^6^^,^^22^

The current study found a statistically significant correlation between the time from diagnosis to HSCT and platelet engraftment. The time from diagnosis to HSCT represents the length of previous treatment. The mean time was 10.7 ± 6.5 months in the entire cohort**.** Based on that fact, we divided the patients into two groups: those whose time was <10 months and those ≥10 months. There was a statistically significant correlation between the length of the previous treatment and platelet engraftment. Patients with previous treatment ≥10 months had significantly faster platelet engraftment than others**.** There was no statistically significant correlation between the length of previous treatment and neutrophil engraftment.

The source of HSCs as a predictive factor for the rate of hematopoietic recovery after HSCT was analyzed in a study by Gonçalves et al., in which a statistically significant correlation between the source of HSCs and the rate of neutrophil and platelet recovery was demonstrated. They found a faster hematologic recovery for neutrophil and platelet engraftment with peripheral blood stem cells.^12^

This study found a statistically significant correlation between the source of HSCs and neutrophil and platelet recovery. Patients for whom peripheral blood was the source of HSCs had significantly faster neutrophil and platelet engraftment than patients whose source of HSCs was BM or a combination of peripheral blood and BM.

## Association between hematopoietic stem cell product quality and engraftment kinetics

The effect of the number of infused CD34^+^ cells/kg on the rate of neutrophil and platelet recovery was tested in the majority of studies; this was proven to be the main predictive factor for engraftment kinetics of autologous HSCs.^6^^,^^9^^,^^10^^,^^13^^,^^21^^,^^23^, ^24^, ^25^, ^26^, ^27^

A CD34^+^ cell dose of 2.5 × 10^6^/kg is accepted as the threshold level for optimum engraftment with higher doses also being associated with faster hematological recovery. On the other hand, it was demonstrated that significantly delayed neutrophil engraftment was observed in patients with doses of CD34^+^ cells lower than 2 × 10^6^/kg.^13^^,^^28^

In a study by Figuerres et al. that included 83 pediatric patients treated with autologous HSCT, the correlation between the numbers of leukocytes/kg, MNC/kg, CD34^+^ cells/kg, Burst-Forming Unit-Erythroid/kg, and CFU-GM/kg and the rate of hematopoietic recovery were tested. They concluded that the number of MNC/kg was the most critical parameter in predicting the time between graft infusion and neutrophil recovery.^29^ A statistically significant correlation between the number of infused MNC/kg and the rate of hematopoietic recovery was demonstrated in a study by Martin et al. Patients who received a higher dose of MNC/kg had better survival and a lower likelihood of relapse. However, there was no statistically significant correlation between the number of infused CD34^+^ cells/kg and the rate of neutrophil and platelet recovery.^30^ The number of MNC/kg did not affect engraftment kinetics and hematopoietic recovery in the current study.

Turk et al. demonstrated a statistically significant correlation between the number of infused CD34^+^ cells/kg and neutrophil engraftment, while this number did not affect platelet engraftment. Patients who received ≥2.5 × 10^6^/kg CD34^+^ cells had better neutrophil recovery than patients who received <2.5 × 10^6^/kg CD34^+^ cells.^19^ The study by Díaz et al. in pediatric patients demonstrated that a dose ≥5 × 10^6^/kg CD34^+^ cells is optimal to ensure rapid neutrophil and platelet recovery.^11^ The effect of the number of CD34^+^ cells/kg, MNC/kg, and CFU-GM/kg on the rate of hematopoietic recovery was tested in this study. A statistically significant correlation existed between the number of CD34^+^ cells/kg and platelet recovery. In contrast, no statistically significant correlation was found between the number of CD34^+^ cells/kg and neutrophil recovery. Patients for whom the number of CD34^+^ cells/kg was ≥6 × 10^6^/kg had a significantly faster platelet recovery than patients for whom that number was <6 × 10^6^/kg.

This study also found a statistically significant correlation between the number of CFU-GM/kg and neutrophil engraftment. Patients for whom the number of CFU-GM/kg was ≥50 × 10^4^/kg had faster neutrophil recovery than patients with a lower number of CFU-GM. That agrees with the work of Diaz et al. but is the opposite of that of Figueres et al. in which the number of CFU-GM/kg was the most crucial parameter predicting the length of time until platelet recovery.^11^^,^^29^

This study has some limitations, considering that it was a retrospective study. The number of patients included was limited within the study period. The heterogenicity of the study population is also a limitation. Future studies should be prospective and multicentric and examine the relationship between specific CD34^+^ subsets (e.g., CD34^+^ 201^+^) in HSC products and engraftment kinetics after autologous HSCT in children.

## Conclusions

The present study established that the patient's age, body weight, diagnosis, source of HSCs, and the number of CFU-GM/kg were predictive factors for neutrophil engraftment kinetics. In contrast, for faster platelet engraftment, the predictive factors were the time from diagnosis to HSCT, the source of HSCs, and the number of CD34^+^ cells/kg. Other factors such as body height, gender, and the number of MNC/kg were not proven to affect neutrophil or platelet engraftment.

## Author contributions

BA, DV, and MK contributed to the study conception and design. Material preparation, collection and analysis of data, and the draft of the manuscript were performed by BA. ZR and MS commented and made suggestions on the first versions of the manuscript. OS corrected the final version of the manuscript. All authors read and approved the final manuscript.

## Conflicts of interest

The authors have no relevant financial or non-financial interests to disclose.
